# SARS-CoV-2 Infection and Hospitalization Among Adults Aged ≥18 Years, by Vaccination Status, Before and During SARS-CoV-2 B.1.1.529 (Omicron) Variant Predominance — Los Angeles County, California, November 7, 2021–January 8, 2022

**DOI:** 10.15585/mmwr.mm7105e1

**Published:** 2022-02-04

**Authors:** Phoebe Danza, Tae Hee Koo, Meredith Haddix, Rebecca Fisher, Elizabeth Traub, Kelsey OYong, Sharon Balter

**Affiliations:** 1 Acute Communicable Disease Control Program, Los Angeles County Department of Public Health, Los Angeles, California.

COVID-19 vaccines are effective at preventing infection with SARS-CoV-2, the virus that causes COVID-19, as well as severe COVID-19–associated outcomes in real-world conditions (*1*,*2*). The risks for SARS-CoV-2 infection and COVID-19–associated hospitalization are lower among fully vaccinated than among unvaccinated persons; this reduction is even more pronounced among those who have received additional or booster doses (boosters) (*3*,*4*). Although the B.1.1.529 (Omicron) variant spreads more rapidly than did earlier SARS-CoV-2 variants, recent studies suggest that disease severity is lower for Omicron compared with that associated with the B.1.617.2 (Delta) variant; but the high volume of infections is straining the health care system more than did previous waves (*5*).*^,^^†^ The Los Angeles County (LAC) Department of Public Health (LACDPH) used COVID-19 surveillance and California Immunization Registry 2 (CAIR2) data to describe age-adjusted 14-day cumulative incidence and hospitalization rates during November 7, 2021–January 8, 2022, by COVID-19 vaccination status and variant predominance. For the 14-day period ending December 11, 2021, the last week of Delta predominance, the incidence and hospitalization rates among unvaccinated persons were 12.3 and 83.0 times, respectively, those of fully vaccinated persons with a booster and 3.8 and 12.9 times, respectively, those of fully vaccinated persons without a booster. These rate ratios were lower during Omicron predominance (week ending January 8, 2022), with unvaccinated persons having infection and hospitalization rates 3.6 and 23.0 times, respectively, those of fully vaccinated persons with a booster and 2.0 and 5.3 times, respectively, those of fully vaccinated persons without a booster. In addition, during the entire analytic period, admission to intensive care units (ICUs), intubation for mechanical ventilation, and death were more likely to occur among unvaccinated persons than among fully vaccinated persons without or with a booster (p<0.001). Incidence and hospitalization rates were consistently highest for unvaccinated persons and lowest for fully vaccinated persons with a booster. Being up to date with COVID-19 vaccination is critical to protecting against SARS-CoV-2 infection and associated hospitalization.

LACDPH conducted a cross-sectional analysis of LAC residents aged ≥18 years with laboratory-confirmed SARS-CoV-2 infection (a positive SARS-CoV-2 result from a nucleic acid amplification or antigen test) during November 7, 2021–January 8, 2022.^§^ Persons were considered fully vaccinated ≥14 days after receipt of the final dose in the primary series of a BNT162b2 (Pfizer-BioNTech), mRNA-1273 (Moderna), or Ad.26.COV2.S (Janssen [Johnson & Johnson]) vaccine and considered unvaccinated if <14 days had elapsed since receipt of the first dose in the primary series of an mRNA or Janssen vaccine or if no matching immunization record was found in CAIR2.^¶^ Fully vaccinated persons who received a booster were considered fully vaccinated with a booster ≥14 days after the date of the booster.** Infections occurring in partially vaccinated persons (persons who had received the first dose in a 2-dose series >14 days earlier, but who were either missing a second dose or <14 days had elapsed since receipt of the second dose) were excluded because of small sample size.^††^ COVID-19–associated hospitalizations were defined as hospital admissions occurring ≤14 days after the first laboratory-confirmed positive SARS-CoV-2 test result (*6*). Whole genome sequencing data from laboratories conducting routine genomic surveillance for LAC were used to calculate weekly variant proportions.^§§^ All available variant data were reported by date of specimen collection and used to assess periods of predominance (>50% of sequenced specimens) for the Delta and Omicron variants.

Demographic and clinical characteristics of SARS-CoV-2 infections were compared by vaccination status using Pearson’s chi-square tests for categorical variables and Kruskal-Wallis tests for medians. P-values <0.05 were considered statistically significant. Age-adjusted rolling 14-day SARS-CoV-2 infection and hospitalization rates and rate ratios among LAC residents aged ≥18 years were estimated by vaccination status using 2019 population estimates and standardized using the year 2000 U.S. standard population.^¶¶^ Analyses were conducted using SAS (version 9.4; SAS Institute) and R (version 3.6.2; R Foundation). This activity was determined by LACDPH’s Institutional Review Board to be a surveillance activity necessary for public health work and therefore did not require Institutional Review Board review.

From mid-August 2021 until the emergence of Omicron in November 2021, nearly 100% of SARS-CoV-2 infections among LAC residents with sequenced specimens were caused by the Delta variant. The earliest known Omicron variant infection in LAC was identified in a specimen collected during the final week of November 2021. As Omicron emerged in LAC, Delta prevalence decreased 95% during the week ending December 11. Omicron became the predominant SARS-CoV-2 variant in LAC during the week ending December 18, accounting for 57% of all sequenced specimens; Omicron prevalence continued to increase, accounting for 99% of all sequenced specimens for the week ending January 8, 2022 (Supplementary Figure, https://stacks.cdc.gov/view/cdc/113859).

Among 422,966 reported SARS-CoV-2 infections in LAC residents aged ≥18 years during November 7, 2021–January 8, 2022, a total of 141,928 (33.6%) were in unvaccinated persons, 56,185 (13.3%) were in fully vaccinated persons with a booster, and 224,853 (53.2%) were in fully vaccinated persons without a booster (Table). Unvaccinated persons were most likely to be hospitalized (2.8%), admitted to an ICU (0.5%), and require intubation for mechanical ventilation (0.2%); these outcomes were less common in fully vaccinated persons with a booster (0.7%, 0.08%, and 0.03%, respectively) and fully vaccinated persons without a booster (1.0%, 0.12%, and 0.05%, respectively) (p<0.001). Deaths were also more likely to occur among unvaccinated persons (0.3%) than among fully vaccinated persons with a booster (0.07%) or without (0.08%) (p<0.001).

**TABLE:** Selected characteristics of cases of SARS-CoV-2 infection in residents aged ≥18 years (N = 422,966), by vaccination status — Los Angeles County, California, November 7, 2021–January 8, 2022*^,^^†^

Characteristic	Vaccination status, no. (column %)		
	Unvaccinated	Fully vaccinated without booster	Fully vaccinated with booster
**Total no. of cases (row %)**	**141,928 (33.6)**	**224,853 (53.2)**	**56,185 (13.3)**
**Median age, yrs (IQR)**	35 (27–48)	36 (27–49)	46 (33–59)
18–29	48,940 (34.5)	74,352 (33.1)	9,523 (16.9)
30–49	61,380 (43.2)	97,771 (43.5)	22,649 (40.3)
50–64	22,338 (15.7)	40,680 (18.1)	14,580 (25.9)
65–79	7,253 (5.1)	9,796 (4.4)	7,960 (14.2)
≥80	2,017 (1.4)	2,254 (1.0)	1,473 (2.6)
**Sex**			
Women	69,382 (48.9)	123,927 (55.1)	30,864 (54.9)
Men	66,163 (46.6)	94,258 (41.9)	23,713 (42.2)
Other or unknown	6,383 (4.5)	6,668 (3)	1,608 (2.8)
**Race/Ethnicity^§^**			
American Indian or Alaska Native	342 (0.2)	426 (0.1)	104 (0.2)
Asian	7,451 (5.2)	18,043 (8.0)	8,341 (14.8)
Black or African American	12,319 (8.7)	13,359 (5.9)	2,632 (4.6)
Hispanic or Latino	42,973 (30.3)	79,198 (35.2)	14,023 (25.0)
Multiple race	494 (0.3)	968 (0.4)	210 (0.3)
Native Hawaiian or Other Pacific Islander	1,429 (1.0)	1,740 (0.7)	608 (1.0)
Other	18,720 (13.2)	32,552 (14.5)	6,808 (12.1)
White	20,529 (14.5)	34,108 (15.2)	12,504 (22.3)
Missing	37,671 (26.5)	44,459 (19.8)	10,955 (19.5)
**Previously documented SARS-CoV-2 infection**	12,360 (8.7)	22,153 (9.9)	3,246 (5.8)
**Hospitalized**	3,989 (2.8)	2,295 (1.0)	413 (0.7)
**Admitted to an intensive care unit**	641 (0.5)	276 (0.12)	47 (0.08)
**Required mechanical ventilation**	256 (0.2)	116 (0.05)	15 (0.03)
**Died**	485 (0.3)	172 (0.08)	40 (0.07)
**Vaccine manufacturer^¶^**			
Johnson & Johnson	—	18,543 (8.2)	4,869 (8.7)
Moderna	—	82,435 (36.7)	19,742 (35.1)
Pfizer-BioNTech	—	123,875 (55.1)	31,574 (56.2)
Median interval between final vaccine dose and infection, days (IQR)**	—	241 (200–271)	49 (31–70)
**Sequencing result available**	7,087 (5.0)	9,663 (4.3)	1,296 (2.3)
**Sequencing result**			
Delta	3,817 (53.9)	3,471 (35.9)	128 (9.9)
Omicron	3,248 (45.8)	6,180 (64.0)	1,164 (89.8)
Other	22 (0.3)	12 (0.1)	4 (0.3)

* Partially vaccinated persons were excluded from this analysis.

^†^ A Pearson’s chi-square test was conducted for categorical variables and Kruskal-Wallis test for medians; p<0.001.

^§^ Race and ethnicity were defined as mutually exclusive categories. Hispanic or Latino includes all persons with ethnicity reported as “Hispanic or Latino” regardless of reported race. “Other” Race/Ethnicity includes persons of multiple races, and persons for whom reported race was “Other.” Missing values were included in statistical testing.

^¶^ The primary vaccine series was used to categorize persons by vaccine manufacturer type regardless of which vaccine manufacturer was received for the booster dose.

** Infection date refers to the earliest of either the date of symptom onset, diagnosis, death, report received, or specimen collection.

During the last week of Delta predominance (week ending December 11), age-adjusted 14-day cumulative incidence and hospitalization rates were highest among unvaccinated persons (443.9 and 45.9 per 100,000 persons, respectively), and lower among fully vaccinated persons with a booster (36.1 and 0.6, respectively) and fully vaccinated persons without a booster (115.9 and 3.6, respectively). As Omicron became predominant, age-adjusted incidence and hospitalization rates increased in all groups, irrespective of vaccination status, compared with rates during the Delta predominant period (Figure 1). As of January 8, 2022, age-adjusted 14-day cumulative incidence and hospitalization rates remained highest among unvaccinated persons (6,743.5 and 187.8 per 100,000, respectively), and lowest among fully vaccinated persons with a booster (1,889.0 and 8.2, respectively) and fully vaccinated persons without a booster (3,355.5 and 35.4, respectively).

**FIGURE 1:**
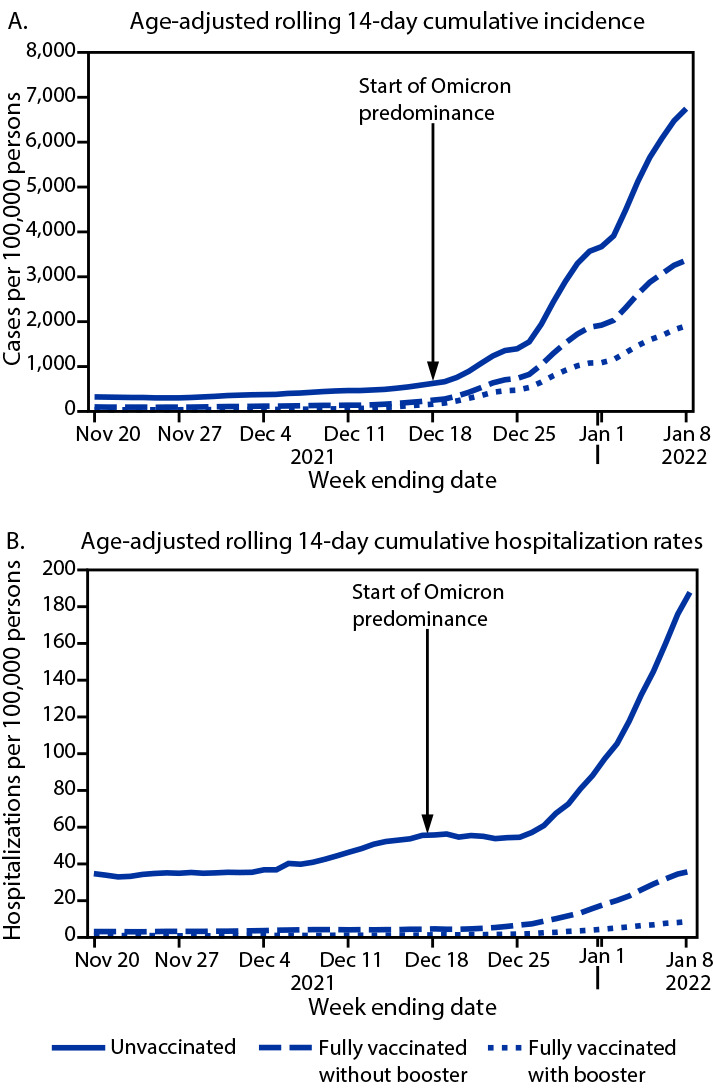
Age-adjusted rolling 14-day SARS-CoV-2 cumulative incidence* (A) and hospitalization rates (B), by vaccination status — Los Angeles County, California, November 7, 2021–January 8, 2022 * Rates were estimated using 2019 population estimates and standardized using the year 2000 standard population.

Overall, during November 7, 2021–January 8, 2022, incidence and hospitalization rates were highest among unvaccinated persons. During the last week of Delta predominance, compared with fully vaccinated persons with a booster, incidence and hospitalization rates among unvaccinated persons were 12.3 and 83.0 times higher, respectively (Figure 2), and compared with rates for fully vaccinated persons without a booster, incidence and hospitalization rates among unvaccinated persons were 3.8 and 12.9 times higher, respectively. As of January 8, 2022, during Omicron predominance, these rate ratios were lower for both comparisons, with infection and hospitalization rates among unvaccinated persons 3.6 times and 23.0 times, respectively, those in fully vaccinated persons with a booster, and 2.0 and 5.3 times, respectively, those in fully vaccinated persons without a booster.

**FIGURE 2:**
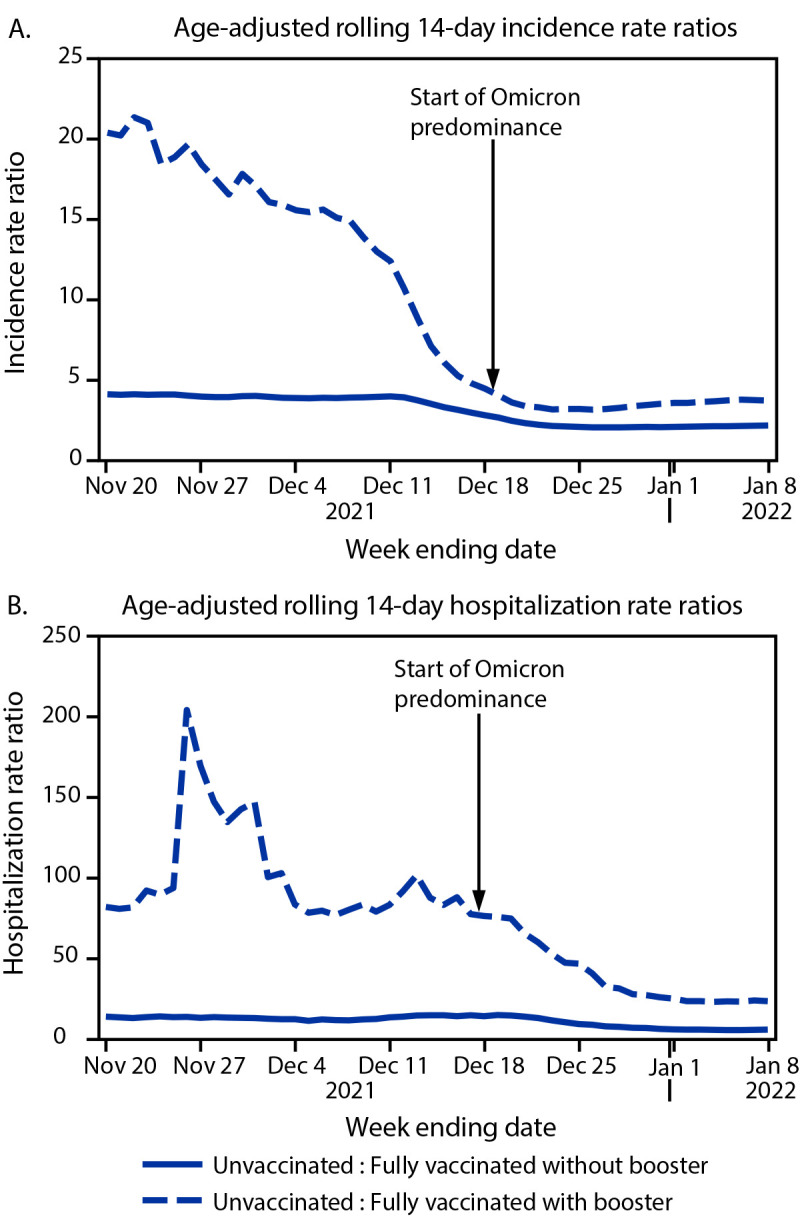
Age-adjusted rolling 14-day SARS-CoV-2–associated incidence rate ratios* (A) and hospitalization rate ratios (B), by vaccination status — Los Angeles County, California, November 7, 2021–January 8, 2022 * Rate ratios were estimated by comparing rates in unvaccinated persons with those in vaccinated persons with and without a booster dose, using 2019 population estimates and standardized using the year 2000 standard population.

## Discussion

During November 7, 2021–January 8, 2022, SARS-CoV-2 infections increased rapidly among LAC adults with the largest increase occurring as Omicron displaced Delta as the predominant circulating variant, leading to decreased incidence and hospitalization rate ratios among unvaccinated persons relative to vaccinated persons with and without a booster. Whereas incidence and hospitalization rates were higher during the Omicron-predominant weeks compared with those during Delta predominance, rate ratios indicated continued protection conferred by vaccine against severe disease, especially among those who had received a booster, although reduced for Omicron compared with Delta. All incidence and hospitalization rate ratios exceeded 1, regardless of predominant variant, indicating that the risks were consistently highest for unvaccinated persons and that COVID-19 vaccines were protective against SARS-CoV-2 infection and COVID-19–associated hospitalization among fully vaccinated persons, and most protective among those with a booster.

Although disease severity appears to be lower for Omicron, a rapid increase in infections during Omicron predominance has resulted in a relatively substantial volume of hospitalizations (*5*). The high volume of hospitalizations during a surge can compound the effects of staffing shortages and staff member burnout, which puts a strain on the health care sector. The rise in hospitalization rates in LAC was most pronounced among unvaccinated persons, whereas hospitalization rates remained lower among those who were fully vaccinated, and lowest among those who had received a booster. Being up to date with COVID-19 vaccinations is a critical component of reducing the strain on health care facilities.

The findings in this report are subject to at least five limitations. First, vaccination data for persons who lived in LAC at the time of their laboratory-confirmed infection, but who were vaccinated outside of California, were unavailable, leading to misclassification of their vaccination status; if vaccinated persons without accessible records were considered unvaccinated, the incidence in unvaccinated persons could be underestimated. Some boosters might have been misclassified as first doses, and the persons receiving these might have been incorrectly classified as partially vaccinated and excluded. Second, aside from age adjustment, it was not possible to control for other factors that are associated with vaccine coverage, such as sex and race/ethnicity. Differences in vaccination and booster coverage by these characteristics, especially if proportionally different from that of SARS-CoV-2 infections, could affect generalizability of these results to LAC and other populations or jurisdictions. Third, the risks for SARS-CoV-2 infection are not equal for everyone; the likelihood of exposure might influence the likelihood of COVID-19 vaccine acceptance and coverage. External risk factors related to the possibility of infection and hospitalization, such as sample characteristics and social determinants of health, are important to consider when interpreting these findings. Fourth, COVID-19–associated hospitalizations were determined based on hospital admission and SARS-CoV-2 test dates alone, potentially leading to the inclusion of incidental positive SARS-CoV-2 test results in patients whose hospitalizations were not caused by COVID-19. Finally, genomic sequencing data were available for only a sample of SARS-CoV-2 specimens and not representative of all infections; however, the variant predominance trends were consistent with what has been reported nationally during these periods.

These findings align with those from recent studies, indicating that COVID-19 vaccination protects against severe COVID-19 caused by SARS-CoV-2 variants, including Omicron (*7*,*8*).*** Efforts to promote COVID-19 vaccination and boosters are critical to preventing COVID-19–associated hospitalizations and severe outcomes. Ongoing COVID-19 surveillance with data linkages to vaccination and SARS-CoV-2 variant genomic sequencing data are critical for monitoring vaccine effectiveness and increased protection from boosters, particularly during the Omicron predominant period.

**Summary**

**What is already known about this topic?**

COVID-19 vaccines are highly effective against severe SARS-CoV-2–associated outcomes, including those caused by the Delta variant.

**What is added by this report?**

As of January 8, 2022, during Omicron predominance, COVID-19 incidence and hospitalization rates in Los Angeles County among unvaccinated persons were 3.6 and 23.0 times, respectively, those of fully vaccinated persons with a booster, and 2.0 and 5.3 times, respectively, those among fully vaccinated persons without a booster. During both Delta and Omicron predominance, incidence and hospitalization rates were highest among unvaccinated persons and lowest among vaccinated persons with a booster.

**What are the implications for public health practice?**

Being up to date with COVID-19 vaccination is critical to protecting against SARS-CoV-2 infection and hospitalization.

## Footnotes

*
https://www.medrxiv.org/content/10.1101/2021.12.30.21268495v1

†
https://www.medrxiv.org/content/10.1101/2022.01.07.22268919v2

§ The population of Los Angeles County residents is based on 2019 population estimates prepared for Los Angeles County Internal Services Department. These population estimates exclude the populations of Pasadena and Long Beach, which have independent public health departments.

¶ Vaccination status was determined using a deterministic and probabilistic matching algorithm to link cases with immunization records in CAIR2 and followed CDC guidelines. https://www.cdc.gov/coronavirus/2019-ncov/vaccines/fully-vaccinated-guidance.html

** CDC recommends that persons aged ≥5 years who are moderately or severely immunocompromised receive a third dose of an mRNA COVID-19 vaccine 28 days after completion of the primary series to enhance the likelihood of mounting an adequate immune response. A booster is recommended for persons aged ≥12 years to protect against waning of vaccine-induced immunity. Recommendations for booster timing and vaccine manufacturer differs by age group and vaccine product received during the primary series (https://www.cdc.gov/coronavirus/2019-ncov/vaccines/recommendations/immuno.html). This analysis does not distinguish between fully vaccinated persons who are immunocompromised and received a booster and fully vaccinated persons who are not immunocompromised and received a booster.

†† Partially vaccinated persons were those who had received the first dose in a 2-dose series ≥14 days earlier, but who were either missing a second dose or <14 days had elapsed since receipt of the second dose. Recipients of the Janssen vaccine <14 days earlier were considered unvaccinated. Partially vaccinated persons accounted for 4% of the sample (18,988). It was also not possible to differentiate between persons who received 1 dose in a 2-dose series (Pfizer-BioNTech or Moderna vaccines) and persons who were misclassified because of missing data.

§§ Whole genome sequencing lineage data were from all sequencing results reported to LACDPH or sequenced after specimens were referred to LACDPH laboratories. Additional variant data were available for specimens sequenced for enhanced Omicron variant surveillance. During the Delta predominant period, sequencing data were available for 20%–25% of SARS-CoV-2 specimens. As infections increased during the Omicron predominant period, capacity for whole genome sequencing did not increase proportionally; sequencing data were available for 1%–18% of SARS-CoV-2 specimens.

¶¶ Adjusted rates were calculated using methods documented by CDC (https://www.cdc.gov/cancer/uscs/technical_notes/stat_methods/rates.htm). A continuity correction was applied to estimates of the unvaccinated population to ensure that ≥5% of the population remains unvaccinated using the methods outlined elsewhere (https://kingcounty.gov/depts/health/covid-19/data/~/media/depts/health/communicable-diseases/documents/C19/calculation-method-technical-appendix.ashx). Adjusted rate ratios were calculated as the cumulative incidence among unvaccinated persons divided by the cumulative incidence among those fully vaccinated with or without booster.

***
https://www.medrxiv.org/content/10.1101/2022.01.11.22269045v1
